# Flow Cytometric Detection of Malignant Blasts in Cerebrospinal Fluid: A Biomarker of Central Nervous System Involvement in Childhood Acute Lymphoblastic Leukemia

**DOI:** 10.3390/biom12060813

**Published:** 2022-06-09

**Authors:** Maria Thastrup, Hanne Vibeke Marquart, Kjeld Schmiegelow

**Affiliations:** 1 Department of Pediatrics and Adolescent Medicine, Rigshospitalet, University of Copenhagen, 2100 Copenhagen, Denmark; maria.thastrup@regionh.dk; 2 Department of Clinical Immunology, Rigshospitalet, University of Copenhagen, 2100 Copenhagen, Denmark; hanne.marquart@regionh.dk; 3 Institute of Clinical Medicine, Faculty of Health and Medical Sciences, University of Copenhagen, 2100 Copenhagen, Denmark

**Keywords:** flow cytometry, cerebrospinal fluid, central nervous system, acute lymphoblastic leukemia, childhood, biomarker

## Abstract

Despite the excellent prognosis for children and adolescents with acute lymphoblastic lymphoma (ALL), the involvement of the central nervous system (CNS) represents a major therapeutic challenge. Patients who develop CNS relapse have a very poor prognosis, and since current methods cannot reliably identify patients with CNS involvement or patients at high risk of CNS relapse, all children with ALL receive CNS-directed treatment. The current golden standard for detecting CNS involvement is the assessment of cytomorphology on cytospin slides of cerebrospinal fluid (CSF). This technique is inadequate due to low sensitivity and reproducibility. Flow cytometric analysis of CSF represent a novel, highly specific and sensitive technique for the detection of leukemic cells in the CNS. In prospective studies, CSF flow cytometry demonstrated two to three times higher rates of CNS involvement at diagnosis of childhood ALL than conventional cytospin, and especially demonstrated superior sensitivity in detecting low-level CNS disease. CNS involvement determined via flow cytometry has been linked to a higher risk of CNS relapse and poor outcomes in several studies. In this review, we discuss the central analytical concepts of CSF flow cytometry and summarize the current evidence supporting the use of flow cytometric detection of malignant blasts as a biomarker of CNS involvement in childhood ALL.

## 1. Introduction

Over the past decades, improved therapy and supportive care have resulted in overall survival rates or 90% or more for patients with childhood (1–17.9 years) acute lymphoblastic leukemia (ALL) [1,2]. However, the dissemination of leukemic cells to the central nervous system (CNS) remains a therapeutic challenge and is associated with an increased risk of CNS relapse [3,4]. The current gold standard for the detection of CNS involvement is microscopic examination of cytomorphology in cytospin preparations of cerebrospinal fluid (CSF). This technique is hampered by low sensitivity [5,6] and reproducibility [7], and most likely underestimates the frequency of CNS leukemia. Today, all patients with ALL receive prophylactic CNS-directed therapy despite its association with serious neurological toxicities [8,9]. Despite aggressive CNS-directed therapy, more than 30% of relapses in childhood ALL involve the CNS [10,11], suggesting that the current treatment is inadequate in high-risk patients. Furthermore, universal CNS-directed therapy leads to overtreatment in low-risk patients and unwarranted side-effects. More sensitive and specific methods for the detection of CNS involvement in childhood ALL are therefore urgently needed.

Recently, flow cytometric analysis of CSF has been proposed as an alternative technique for the detection of CNS leukemia. Flow cytometry can detect malignant cells in the CSF with high sensitivity and reproducibility and thus holds potential for directing risk-adapted CNS-directed therapy in childhood ALL. The purpose of this article is to review central analytical concepts in CSF flow cytometry and the current evidence supporting the use of the measured blast count as a prognostic biomarker in childhood ALL.

## 2. CNS Involvement in Childhood ALL

Disseminated leukemic cells predominantly reside within the leptomeninges that cover the surface of the brain and spinal cord and only invade the brain parenchyma in late-stage disease [12]. The leukemic cells are found in the subarachnoid space, where they adhere to the meningeal membranes and/or circulate in the CSF [13]. Since leukemic cells are found in the CSF, obtaining a sample of CSF via a lumbar puncture and examining the CSF for the presence of leukemic cells is a straightforward way of diagnosing CNS involvement. However, at present, the relationship between adherent and circulating leukemic cells in the leptomeninges has not been clarified, and it is not known if there are individual differences in leukemic cell distributions. The circulating cells may represent a subpopulation of leukemic cells with specific properties, or they may simply be apoptotic cells shed from adherent leukemic cell colonies.

CNS involvement is associated with a number of high-risk characteristics, including infant age, T-cell lineage, hyperleukocytosis, and specific chromosomal aberrations in B-ALL, such as rearrangements of the *KMT2A* gene, t(9;22)[BCR-ABL1] and t(1;19)[TCF3-PBX1] [14]. Leukemic cells are disseminated to the CNS early in the course of the disease, and with current diagnostic techniques CNS involvement is detected in approximately 10% of patients at diagnosis [3,4,15,16]. However, due to the limitations of the current diagnostic techniques, the true frequency of CNS involvement is probably higher. This is supported by the fact that even patients without cells in their CSF often have significant CNS infiltration on post-mortem brain biopsies [12]. CNS involvement at diagnosis is associated with inferior survival and a higher rate of CNS relapse [4]. However, most CNS relapses are seen in patients who were CNS-negative at diagnosis [11,15], underscoring the shortcomings of current CNS classification. In addition, not all patients who are CNS-positive at diagnosis develop CNS relapse. Due to the poor prognostic value of current CNS classifications, all patients with ALL receive CNS-directed therapy, consisting of intrathecal chemotherapy, systemic high-dose chemotherapy and, in some cases, cranial irradiation [14]. These treatments are associated with acute and chronic neurotoxicity, leading to a greater risk of permanent cognitive and neuroendocrine impairments [8,9,17].

## 3. Current Biomarkers of CNS Involvement

Today, the conventional method for diagnosing CNS involvement in childhood ALL is based on cytomorphology [5,6]. After obtaining a CSF sample via a lumbar puncture, the cells from a small aliquot of CSF are spun onto a microscope slide via low-speed centrifugation. After May–Grunwald–Giemsa staining, the cells are examined under a microscope by an experienced pathologist and lymphocytes present on the cytospin are classified as malignant or non-malignant [18]. Unfortunately, this method is challenged by both low sensitivity (~50%) [5,6] and low reproducibility (~50%) [7].

Microscopy-based analysis has low sensitivity in the detection of rare cells, such as malignant blasts in the CSF of patients with disseminated CNS disease [5,19]. Often only a few milliliters of CSF are used for preparing cytospin [19], and with small sample volumes, the scarce leukemic blasts might be missed. Using higher volumes of CSF increases the sensitivity of the analysis, and previous studies have recommended the collection of between 8.5 and 10.5 mL of CSF to obtain >90% sensitivity [19,20]. However, sampling of such large volumes will not be feasible for all children. Furthermore, several studies have shown that leukocytes in CSF rapidly decay ex vivo. Only 1–2 h after sample collection, the number of lymphocytes in the CSF was reduced by 30%–40% [21,22,23]. If cytospin samples are not processed immediately, the degradation of blasts will further reduce cytospin sensitivity and lead to the underestimation of CNS leukemia based on CSF cytology.

As part of the immune surveillance in the CNS, the CSF also contains normal lymphocytes, especially T-lymphocytes [24]. Activated lymphocytes may be morphologically atypical and can be difficult to distinguish from malignant blasts in cytospin, especially in cases with low-level CNS disease [25,26,27]. Difficulties in discriminating normal and malignant lymphocytes can lead to both false negatives and false positives. The level of reproducibility in the cytospin results from two different laboratories analyzing the same CSF sample was only 54% in a recent study [7], which underscores the highly subjective nature of cytomorphology analysis.

CNS leukemia is diagnosed based on CSF cytospin results, together with leukocyte counts and, in some cases, observation of the clinical signs of CNS leukemia. CNS leukemia is diagnosed if at least one blast cell is observed on cytospin. Patients without CNS involvement are classified as CNS1 (<5 leukocytes/µL CSF with no blasts on cytospin) and patients with CNS involvement as CNS2 (<5 leukocytes/µL CSF with blasts on cytospin) or CNS3 (≥5 leukocytes/µL CSF with blasts on cytospin and/or clinical signs of CNS involvement), depending of the level of CNS infiltration [14]. Clinical signs of CNS leukemia include various neurological symptoms, such as cranial nerve palsy, intracranial mass on MRI or retinal involvement. CNS3 disease has been linked to an increased risk of CNS relapse and poor outcomes in several studies [4,28,29]; however, the association between CNS2 and later CNS relapse is more inconsistent [4,15,28]. These differences can probably be explained by inconsistent cytospin procedures between studies, rather that clinical differences, since large variations in CNS2 rates have been observed. Even though CNS3 and in some studies CNS2 have been reported as prognostic factors of CNS relapse, most CNS relapses occur in CNS1 patients, which underscores the need for better biomarkers for CNS leukemia [11,15].

## 4. Central Methodological Concepts of CSF Flow Cytometry

Recently, multicolor flow cytometric analysis of CSF has been proposed as a more sensitive method for detecting CNS involvement in hematological malignancies. When performing immunophenotyping via flow cytometry, cells are stained with fluorochrome-conjugated antibodies against multiple markers, and the fluorescent properties of each individual cell are determined with a flow cytometer. Malignant cells are identified based on aberrant expression patterns not observed in non-malignant cells, making this technique highly specific [30]. Flow cytometry can rapidly and precisely quantify the expression of multiple cell surface molecules, leading to a much higher sensitivity than conventional cytology. In addition, the concordance between laboratories analyzing the same sample for malignant cells is more than 90% for CSF flow cytometry [7].

As part of the diagnostic workup for a patient with suspected leukemia, flow cytometric analysis is performed on bone marrow cell preparations with several pre-determined antibody panels. The lymphoblasts are identified based on their abnormal expression of these markers, a so-called leukemia-associated immunophenotype [30]. The flow cytometric results are used to confirm the diagnosis and classify the immunophenotype (B- or T-lineage) based on the expressed markers. During the treatment of patients with leukemia, minimal residual disease (MRD) in the bone marrow is monitored via flow cytometry and using this technique it is possible to detect malignant cells representing as little as 0.01% of normal bone marrow cells (one cell in 10,000) [30]. The panels developed for bone marrow MRD analysis have, with a few adjustments, been successfully applied for the detection of leukemic cells in the CSF of children with ALL (Table 1). Immunophenotypic identification of leukemic blasts in the CSF is relatively easy, since the malignant cells have an immature phenotype which is normally not present in the CNS [7]. The number of colors applied for CSF flow cytometry over the past 10+ years varies from two to eight. Using a panel with at least six colors is recommended to ensure the correct identification of leukemic cells in childhood ALL [30]. In addition, a T-cell marker such as CD3, CD7 and/or CD8 should be included in the B-lineage panel to exclude the possibility that the identified blasts are normal T-lymphocytes, which are found in small numbers in the CSF [31,32,33].

One of the challenges of CSF flow cytometric analysis is the paucity of malignant cells in the CSF. For bone marrow MRD analysis via flow cytometry, a requirement of a minimum of 10 identified blasts has been applied to ensure that the events represent an actual leukemic cell population [34]. Similar criteria have been applied for CSF flow cytometry in childhood ALL, with the minimum number of phenotypically aberrant cells varying from 5 to 30 [7,33,35,36] (Table 1). Other studies have specified that a “cluster” of events with the same immunophenotype as leukemic blasts at diagnosis designates a positive sample, without specifying the minimum number of events in the cluster [37]. Since leukemic cells in CSF are rare, the sensitivity of CSF flow cytometry is dependent on the volume of CSF analyzed. The sensitivity of both flow cytometry and cytospin is dependent on the analyzed sample volume and the absolute number of cells. However, smaller volumes and lower numbers of cells can be used for CSF flow cytometry due to the higher sensitivity of the analysis. The minimum sample volume used for CSF flow cytometry was between 1 and 3 mL (or more) in childhood ALL studies [35,36,38,39,40]; however, in some studies the CSF volume was not reported [7,33,37,41]. A minimum volume of 1–2 mL has been recommended for CSF flow analysis in ALL [31,32], but the analysis of larger sample volumes will further increase sensitivity.

Flow cytometry requires intact cells for analysis, and therefore the sensitivity of the analysis will also decrease with the degradation of lymphocytes in the CSF. However, the cells can be fixed prior to flow cytometry, e.g., via the collection of CSF directly into specialized Transfix tubes containing an optimized formaldehyde solution with EDTA for the preservation of CSF cells [42]. Our group has shown that flow cytometric analysis of CSF preserved with Transfix can be delayed for up to 48–72 h, whereafter the sensitivity decreases due to a reduced signal from the most commonly used markers [32]. This allows for the shipment of samples and centralized analysis, e.g., at a national MRD laboratory with the resources for the detection of malignant blasts with atypical marker profiles. Previous research has also shown that the addition of serum-containing medium to CSF preserves leukocytes for up to 5 h [23], and this approach has also been used in a few studies [7]. CSF flow studies not using stabilization generally limit the timeframe from sampling to processing or analyzing (between 2 and 24 h) [7,35,37,38]; however, even within a few hours a large fraction of the lymphocytes are degraded, which reduces the sensitivity of the analysis.

**Table 1:** Flow cytometric methodology for CSF analysis in childhood ALL studies. CSF: cerebrospinal fluid; FCM: flow cytometry; BM: bone marrow.

Study	Number of Colors	Panel Selection	Antibody Panel(s)	CSF Stabilization	Sample Volume	Criteria for FCM+	Comments
Sayed et al., 2009 [38]	2–3	Predetermined panels based on immunophenotype.	CD45/CD14; CD4/CD8/CD3; cyTDT/CD10/CD19; kappa/lambda/CD19; CD34/CD19; CD33/CD19	No stabilization. Samples processed within 2 h.	>1 mL	Not reported.	FCM could not be performed for samples with less than one cell/10 µL of CSF.
Martinez-Laperche et al., 2013 [39]	6	Predetermined panels based on immunophenotype.	B-lineage: CD22/CD34/7AAD/CD10/CD19/CD45; T-lineage: CD7/CD8/ 7AAD/CD4/CD3/CD45	Collection in Transfix tubes.	1–8 mL	>0.1% blasts with same immunophenotype as leukemic blasts in BM or blood at diagnosis.	Minimum of 50 viable events for sample to be considered evaluable.
Ranta et al., 2015 [41]	3–8	Predetermined panels based on immunophenotype.	Various panels including the following markers: CD14, GPA, CD45, CD19, CD33, CD3, CD10, CD34, Tdt, CD7, cytCD3, CD61, kappa, lambda, CD20, CD38, CD117, CD4, CD8, CD5, CD22, CD99, HLA-DR, CD2, CD1a	Samples processed immediately or stabilized with Transfix and analyzed the next morning.	50–100 µL per antibody combination.	Not reported.	Minimum of 50 viable events for sample to be considered evaluable.
Cancela et al., 2017 [35]	4	Predetermined panels based on immunophenotype.	Mature T-cells: CD3/CD8/ CD45/CD4; B-lineage: CD10/CD34/CD45/ CD19; T-lineage: CD1a or CDRαb/ CD7/CD45/CD3	No stabilization. Samples processed within 8 h.	~3 mL	Minimum 10 blasts with same immunophenotype as leukemic blasts in BM or blood at diagnosis.	
Gabelli et al., 2019 [37]	8	Not reported.	Not reported.	No stabilization. Samples processed within 24 h.	Not reported.	Cluster of events with same immunophenotype as leukemic blasts in BM or blood at diagnosis.	
Popov et al., 2019 [36]	4–6	Predetermined panels based on immunophenotype.	B-lineage: CD19/CD10/CD34/ CD45; T-lineage: CD7/CD3/ CD5/CD2/CD99/CD45	Not reported.	≥1 mL	Minimum 30 phenotypically aberrant cells.	
Thastrup et al., 2020 [33]	6–7	Predetermined panels based on immunophenotype.	B-lineage: CD3/CD10/CD19/CD20/CD34/CD38/CD45; T-lineage: CD3/CD4/CD7/CD8/CD45/CD56.	Collection in Transfix tubes.	Not reported.	Minimum 10 phenotypically aberrant cells.	
Torkashvand et al., 2020 [40]	Not reported.	Predetermined panels based on immunophenotype.	Panels not reported. Individual markers include: CD2, CD3, CD7, CD5, CD10, CD19, CD20, CD22, HLA-DR, CD45 and CD34.	Collection in fixative medium with EDTA anticoagulant. Transported at 4 °C and processed within 2 h.	~1.5 mL	Not reported.	
De Haas et al., 2021 [7]	6	Predetermined panels based on immunophenotype.	B-lineage: CD58/CD19/CD45/CD10/CD22/CD34 or TdT/CD19/CD45/CD10/CD38/CD20; T-lineage: CD2/CD16+56/CD45/CD7/CD5/mCD3 or CD99/CD16+56/CD45/CD7/CyCD3/mCD3	No stabilization. Local analysis performed within a few hours. Samples for centralized analysis diluted 1:1 in sterile medium and processed within 24 h.	Not reported.	Cluster of events with same immuno-phenotype as leukemic blasts in BM or blood at diagnosis. Minimum of five positive cells in at least one tube.	FCM at both local immunophenotyping laboratory and central reference laboratory.

## 5. Frequency of CSF Involvement by CSF Flow Cytometry

In childhood ALL, several studies have used flow cytometry for the detection of leukemic blasts in CSF (Table 2). CNS involvement determined via flow cytometry has been most intensively studied at initial diagnosis and upon diagnostic lumbar puncture, flow positivity was generally reported to be two to three times higher than the rates observed with cytospin. In studies with unselected patient cohorts (e.g., no positive or negative selection of patients with neurological symptoms or high-risk characteristics) CSF flow positivity at diagnosis ranged from 16.3% to 40.5% compared to between 2.8% and 18.1% for cytospin [33,35,36,37,39,41]. In studies reporting the number of blasts, samples that were positive based on both flow and cytospin results had higher blasts levels than patients who were only positive based on flow results [33,36], illustrating that CSF flow is able to detect low-level CNS disease that is missed by cytospin analysis. One study had a very high frequency of CNS involvement determined by cytospin (51%), which was higher than the frequency determined by flow cytometry (22.7%) [7]. In this study, each cytospin slide was examined by two experienced technicians and the sample was classified as CNS2 if at least one of the observers classified one cell as malignant. In fact, 60% of CNS2 cases in the study could be attributed to the presence of only one or two blasts [7]. Therefore, the high cytospin frequency was attributed to a high false-positive rate due to the insufficient specificity of malignant blasts versus normal lymphocytes.

In several studies, samples with discrepant CSF flow and cytospin results were reported, i.e., cases that are positive based on cytospin analysis but not based on flow analysis. In all studies, the discrepant results affected less than 5% (range 0.5%–4.4%) of samples [33,35,36,37,38,41]. This discrepancy was attributed to (1) the long time from sampling to analysis of CSF flow samples, leading to cell degradation and reduced sensitivity; (2) few positive blasts determined by CSF flow not meeting the study criteria for flow positivity (e.g., less than 10 blasts in total); and (3) few blasts present on cytospin, potentially representing a false positive sample. In rare T-ALL cases it might not be possible to find a suitable marker combination to identify the leukemic blasts in the CSF via flow cytometry if the markers are not adapted according to the bone marrow immunophenotype.

Two studies also collected CSF samples for flow cytometric analysis at relapse. In one of these studies (*n* = 17), three patients (17.6%) were identified with CNS involvement via cytospin analysis compared to two patients via flow cytometry (11.7%) [35]. In the other study (*n* = 13), only patients with isolated bone marrow relapse were included, and more than half of these seemingly CNS disease-free patients were positive via CSF flow cytometry [37]. Two studies also examined follow-up samples after initial diagnosis and/or relapse. None of the follow-up samples collected after initial diagnosis were positive via cytospin, whereas 3.6%–7.2% of samples were positive via CSF flow cytometry [37,39]. After relapse, only 3.1% of samples were positive via cytospin compared to 20.4% via flow cytometry [37]. In our Nordic cohort, we observed that 7.5% of patients at initial diagnosis, who were positive prior to treatment, were still positive at treatment day 15 [33]. Thus, CSF flow cytometry seems to be a promising tool to identify a subgroup of patients with slow CNS clearance.

## 6. Prognostic Significance of CSF Flow Cytometry

Accumulating evidence supports the prognostic significance of CSF flow cytometry in childhood ALL. Out of six studies reporting relapse or survival estimates for flow-positive versus flow-negative patients (Table 3), five studies found a significantly higher risk of relapse and/or poorer survival among flow-positive patients [7,33,35,36,41]. In the latter study, the 3-year cumulative incidence of relapse was higher in the flow-positive group, but did not reach statistical significance, most likely due to the small patient cohort [39]. Interestingly, two studies also confirmed this association specifically for flow-positive patients with low-level CNS disease, i.e., classified as CNS1 or CNS2 via conventional cytospin analysis [7,33].

Since CNS involvement in childhood ALL is more prevalent among patients with other high-risk factors, it is important to confirm that the prognostic significance of flow positivity is an independent risk factor for relapse. In a large study (*n* = ~700 patients) by the NOPHO group, multivariate analysis including known risk factors (sex, age, white blood cell count, immunophenotype and MRD level at day 29) confirmed that CNS positivity via flow analysis was an independent risk factor for relapse (HR 2.2 (95% CI 1.0–4.7)) [33]. A few studies also determined the blast levels via flow cytometry and patients with high blast levels had a higher risk of relapse compared to patients with low-level CNS involvement [33,36]. Multivariate analysis confirmed that a high blast level (≥20 blasts/mL) determined via flow cytometry was an independent risk factor for relapse (HR 4.65 (95% CI 1.3–16.6)) [36].

A traumatic lumbar puncture (TLP) refers to artificial blood contamination of the CSF (≥10 erythrocytes per µL CSF) that can occur during the puncture procedure. TLP has previously been associated with blasts in the CSF and an increased risk of CNS relapse in childhood ALL [15,28,43,44]. Currently, the biological relationship between iatrogenically introduced leucocytes and the risk of relapse has not been clarified. However, in a recent study it was reported that TLP at diagnosis was only associated with a higher risk of relapse in patients if the presence of leukemic blasts in the CSF was confirmed by flow cytometry [33]. Thus, CSF flow cytometry seems to be a useful technique for the reliable classification of lymphoblasts and has been incorporated into the current European ALLTogether1 treatment protocol (NCT04307576) for the confirmation of CNS involvement in the case of a TLP.

Four studies showed that flow cytometry could detect low-level CNS disease in follow-up samples [33,35,37,39], and in these two studies the prognostic significance of persistent malignant blasts during treatment was investigated. In the first study, subclinical levels of CNS involvement during treatment detected via flow cytometry were associated with a significantly higher 3-year cumulative risk of relapse (28.3% vs. 1.3%, *p* < 0.001) [39]. In the second study, patients with high blast level at initial diagnosis (≥20 blasts/mL) had a significantly higher cumulative incidence of relapse if the second sample was also flow-positive compared to patients who cleared their CSF of blasts after initial treatment (71% vs. 33%, *p* = 0.027) [36]. The same tendency was observed for patients who were positive at initial diagnosis with a second positive sample irrespective of the initial blast level, but the difference did not reach statistical significance (50% vs. 29%, *p* = 0.173) [36].

## 7. Perspectives

The current literature clearly shows that CSF flow cytometry markedly increases the detection rates of CNS involvement in childhood ALL compared to cytospin, supporting the proposition that CSF flow cytometry should be incorporated as part of routine diagnostics to increase the sensitivity of CNS leukemia detection. Several studies have also reported a small fraction of samples (<5%) with discrepant cytospin and flow cytometry results, i.e., samples that were only classified as CNS-positive via cytospin analyses, which might largely be attributed to technical issues in relation to sampling and transportation.

Increasing evidence supports that CNS involvement determined via CSF flow cytometry is associated with poorer prognosis and a higher risk of CNS relapse. However, the question of how this knowledge should be translated into risk stratification and the allocation of CNS-directed treatment currently remains unanswered. Not all patients who are CSF flow-positive at diagnosis develop relapse and further research is needed to clarify if CNS involvement at very low levels translates into a higher risk of relapse to determine which patients will benefit from more intensive CNS-directed therapy. Furthermore, it is also important to determine if negative CSF flow cytometry at diagnosis can identify very-low-risk patients in whom CNS-directed therapy can be reduced or even omitted.

High-sensitivity monitoring of CNS involvement during treatment and the question of how the slow clearance of leukemic blasts correlates to outcomes represent interesting prospects related to the use of CSF flow cytometry in childhood ALL. If slow clearance indicates a higher risk of treatment failure or CNS relapse, this technique could be used for the monitoring of treatment and risk assignment by means of “CSF MRD”, similarly to bone marrow MRD, performed as part of current treatment protocols. However, the existing data are scarce, and this concept should be explored in larger patient cohorts to determine if low-level CNS involvement during treatment signifies high-risk patients who require intensified CNS-directed therapy.

Despite the large body of data supporting the superior sensitivity of CSF flow cytometry compared to cytospin, the current cytospin methodology could be significantly improved. Increasing the volume of CSF used for cytospin, reducing the time to sample processing or developing methods for the better preservation of cells compatible with May–Grunwald–Giemsa staining will improve the sensitivity of cytospin analysis. However, discriminating between atypical lymphocytes and malignant blasts will still be difficult when few blasts are present, and in terms of specificity, CSF flow analysis remains superior to cytospin. A drawback of flow cytometry is that this technique is more expensive than cytospin in terms of reagents and instrumentation, and therefore less accessible in lower-income countries. Both techniques should therefore be further developed to ensure access to CNS leukemia diagnostics in all parts of the world.

ALL is the childhood malignancy in which CSF flow cytometry has been most extensively studied. However, this technique also holds potential for the detection of disseminated malignant cells in other hematological and solid cancers characterized by CNS involvement. CSF flow cytometry has demonstrated promising results in adults with acute myeloid leukemia (AML) [45,46] and non-Hodgkin lymphoma (NHL) [47,48,49], but until now remains largely unexplored in childhood AML and NHL.

## Figures and Tables

**Table 2:** Frequency of CNS involvement determined via flow cytometry and cytomorphology analysis in childhood ALL studies. * Twelve patients examined due to presence of neurological symptoms. Thirty-three patients examined as part of routine diagnostic work-up. Fifteen samples not analyzed via FCM due to insufficient cell numbers. ** Only patients who were FCM+ at diagnosis were sampled during follow-up. *** Patients with neurological symptoms or high-risk characteristics excluded from study. LP: lumbar puncture; FCM: flow cytometry; CM: cytomorphology; BM: bone marrow.

Study	Number of Patients	Number of Samples	Diagnosis				Relapse			
			Initial LP		Follow-Up LPs		Initial LP		Follow-Up LPs	
			FCM+	CM+	FCM+	CM+	FCM+	CM+	FCM+	CM+
Sayed et al., 2009 * [38]	45	45 samples collected at initial diagnosis	46.6% (21/45)	22.2% (10/45)	-	-	-	-	-	-
Martinez-Laperche et al., 2013 [39]	108	990 samples collected at diagnosis and during follow-up	27.8% (30/108)	2.8% (3/108)	7.2% (63/882)	0% (0/882)	-	-	-	-
Ranta et al., 2015 [41]	214	214 samples collected at diagnosis	17.3% (37/214)	9.8% (21/214)	-	-	-	-	-	-
Cancela et al., 2017 [35]	67	72 samples collected at diagnosis and relapse	16.3% (9/55)	0% (0/55)	-	-	11.7% (2/17)	17.6% (3/17)	-	-
Gabelli et al., 2019 [37]	97	1050 samples collected at diagnosis or isolated BM relapse and during follow-up	40.5% (29/84)	8.3% (7/84)	3.6% (31/855)	0% (0/855)	53.8% (7/13)	0% (0/13)	20.4% (20/98)	3.1% (3/98)
Popov et al., 2019 [36]	155	155 samples collected at diagnosis	37.4% (53/155)	18.1% (28/155)	-	-	-	-	-	-
Thastrup et al., 2020 ** [33]	673	936 samples collected at diagnosis and during follow-up	25.4% (171/673)	13.4% (90/673)	37.4% (64/171)	-	-	-	-	-
Torkashvand et al., 2020 *** [40]	30	30 samples collected at diagnosis	16.7% (5/30)	0% (0/30)	-	-	-	-	-	-
De Haas et al., 2021 [7]	255	255 samples collected at diagnosis	22.7% (58/255)	51.0% (130/255)	-	-	-	-	-	-
		**Average % (Total number of samples)**	**27.9%** **(413/1619)**	**17.9%** **(289/1619)**	**8.3%** **(158/1908)**	**0%** **(0/1737)**	**30.0%** **(9/30)**	**8.8%** **(3/30)**	**20.4%** **(20/98)**	**3.1%** **(3/98)**

**Table 3:** Prognostic significance of leukemic cells detected via CSF flow cytometry at initial diagnosis in childhood ALL studies. Only studies reporting estimates of cumulative incidences of relapse or survival rates are included in the table. Reported patient numbers specify newly diagnosed patients included in the study. * Data from FCM+ and/or CM+ patients. ** Data from CNS2 patients. FCM: flow cytometry; CIR: cumulative incidence of relapse; OS: overall survival; EFS: event-free survival; IQR: interquartile range; RFS: relapse-free survival.

Study	Number of Patients	Median Follow-Up (Range)	Relapse				Survival			
			Relapse Estimate	FCM+	FCM−	*p*-Value	Survival Estimate	FCM+	FCM−	*p*-Value
Martinez-Laperche et al., 2013 [39]	108	Not reported.	3 year CIR	10.7%	6.9%	0.648	3 year OS	96.6%	96.7%	0.82
Ranta et al., 2015 [41]	214	Not reported.	5 year CIR	29%	7%	0.028	5 year EFS	73% *	86%	0.034
Cancela et al., 2017 [35]	55	24.8 months (28 days–43 months)	-	-	-	-	3 year OS	35.6%	75.4%	<0.0001
Popov et al., 2019 [36]	155	Not reported.	7 year CIR	25%	13%	0.017	-	-	-	-
Thastrup et al., 2020 [33]	673	2.7 years (IQR 1.5–3.9)	4 year CIR	16.5%	5.6%	< 0.001	4 year EFS	80.4%	92.7%	<0.001
De Haas et al., 2021 [7]	255	Not reported.	-	-	-	-	5 year RFS	87.9% **	100% **	0.003
